# EFFECTS OF FASTING STATUS ON PLASMA ENDOCRINE BIOMARKERS IN OLDER ADULTS

**DOI:** 10.1093/geroni/igae098.2622

**Published:** 2024-12-31

**Authors:** Erik Knight, Robert Tennyson, Molly Wright, Jennifer Graham-Engeland, Christopher Engeland

**Affiliations:** University of Colorado Boulder, Boulder, Colorado, United States; The Pennsylvania State University, University Park, Pennsylvania, United States; The Pennsylvania State University, University Park, Pennsylvania, United States; Pennsylvania State University, University Park, Pennsylvania, United States; The Pennsylvania State University, University Park, Pennsylvania, United States

## Abstract

Accurate biomarker measurement is critical for understanding biopsychosocial and cognitive aging. However, biomarker measurement often relies on fasting samples, whereas cognitive testing is typically conducted non-fasting. The effects of eating on endocrine levels were tested in a well-controlled experiment. Older adults (N=30, mean 67.5 years, 70% women) each attended two, morning laboratory sessions with four, hourly blood draws after >8 hours fasting. A meal was provided after the second or fourth blood draw (session order counter-balanced). Eight hormones were assayed via LC/MS; bioavailable hormones were estimated using SHBG (EIA assays). Bayesian multilevel models (minimally informative priors, Stan MCMC sampler) compared pre-prandial (6 samples from both visits) vs. post-prandial (2 samples) concentrations (covariates: time-of-day, visit order, age, gender, BMI). These models provided decisive evidence for eating increasing cortisol and reducing total testosterone and estrone; weak evidence for increasing corticosterone and DHEAS and decreasing progesterone, free testosterone and estrone. Decisive evidence was observed for cortisone, and total and free estradiol being unaffected by eating. Exploratory analyses of gender moderation provided decisive evidence that effects of eating on total testosterone were most strongly observed among men, although the pattern was similar for women. These findings indicate that cortisol, total testosterone, and total estrone are susceptible to changes due to eating among older adults, suggesting careful consideration related to diet/fasting should be made in studies incorporating these biomarkers. Cortisone and estradiol appear relatively unaffected by eating; further research is necessary for all remaining biomarkers that had weak evidence of change.

